# The importance of cardiorespiratory fitness and sleep duration in early CVD prevention: BMI, resting heart rate and questions about sleep patterns are suggested in risk assessment of young adults, 18–25 years: The cross-sectional lifestyle, biomarkers and atherosclerosis (LBA) study.

**DOI:** 10.1186/s12889-020-09801-3

**Published:** 2020-11-16

**Authors:** Maria Fernström, Ulrika Fernberg, Anita Hurtig-Wennlöf

**Affiliations:** 1 Åstrand Laboratory of Work Physiology, The Swedish school of sport and health science, GIHLidingövägen 1, Box 5626, 114 86 Stockholm, Sweden; 2 School of Health Sciences, Faculty of Medicine and Health, Örebro University, 701 82 Örebro, Sweden

**Keywords:** Young adults, Cardiorespiratory fitness, Sleep habits, Resting heart rate, BMI and cardiovascular disease

## Abstract

**Background:**

Cardiorespiratory fitness (CRF) and sleep habits are lifestyle factors with potential to prevent cardiovascular disease (CVD). CVD is the leading cause of death worldwide. It is therefore important to establish a healthy lifestyle at a young age. In the Lifestyle, Biomarkers and Atherosclerosis (LBA) study we have examined 834 healthy non-smoking adults, aged 18–25 years. The general purpose of the LBA study was to study the effect of lifestyle on traditional biomarkers known to influence CVD risk. The aims of the present study were to evaluate sleep habits of young adult women and men participating in the LBA study, and to compare the importance of sleep and other lifestyle habits on clinically relevant biomarkers for CVD. An additional aim was to find easy and reliable non-invasive biomarkers to detect young adults with increased risk of developing CVD later in life.

**Methods:**

The participants had previously been examined for lifestyle factors, biomarkers and CVD risk score. They filled in a validated computerized questionnaire about their general physical and mental health. The questionnaire included questions on sleep duration and experienced quality of sleep.

**Results:**

In total 27% of the young adult participants reported difficulties falling asleep or experienced troubled sleep with frequent awakenings per night. The experienced troubled sleep was not related to a higher CVD risk score, but sleep quality and duration were correlated. Shorter sleep duration was significantly associated to higher body mass index (BMI), body fat (%), homeostasis model assessment of insulin resistance (HOMA-IR) and CVD risk. The modifiable lifestyle factor with the highest odds ratio (OR) for CVD risk was CRF. Sleep duration was the second most influential lifestyle factor, more important than moderate- and vigorous physical activity (MVPA) and food habits. Correlations between CRF and heart rate (HR), (*P* < 0.01) and HOMA-IR and BMI (P < 0.01) were observed, indicating that BMI and resting HR in combination with questions about sleep patterns are easy and reliable non-invasive biomarkers to detect young adults who need counselling on a healthy lifestyle.

**Conclusion:**

Decreased sleep duration in combination with decreased CRF, in young adults, is a serious health issue.

## Background

Cardiorespiratory fitness (CRF), moderate- and vigorous physical activity (MVPA), good dietary and sleep habits are major modifiable lifestyle factors known to have the potential of preventing or delaying cardiovascular disease (CVD) [1–3]. CVD is the leading non-communicable cause of death worldwide [4], and is a disease that starts already in childhood, progressing silently over many years [5, 6]. Despite this, and the severity of the disease, knowledge regarding behavioural risk factors is limited in young adults [7].

Sleep habits have lately become increasingly recognized as an important modifiable lifestyle factor for young adults. Sleep duration is especially important in this age group, and might impact on long-term health and risk for CVD [8]. It is known that many young adults seek help for complaints or conditions that are related to poor sleep, conditions that, if not resolved, may result in long-term sleep problems [9]. It has also recently been shown that new habits and use of technical equipment, such as computers and mobile phones, prevent young adults from obtaining sufficient sleep. Observed insufficient sleep duration was linked to hypertension, insulin resistance, type 2 diabetes and weight gain [10]. Previous reviews and meta-analyses have reported associations between sleep duration and obesity. The majority of reviews support the conclusion by Nielsen, et al. that a short sleep duration is consistently associated with development of obesity in children and young adults, but not consistently so in older adults [8]. The relationship between insomnia and CRF have been studied and the authors conclude that insomnia may require a specific focus in health campaigns addressing reduced CRF and risk of CVD [11]. It has also been shown that sleep deprivation has a stronger effect on mood impairment in young adults compared to in older adults [12] making it an important factor for the well-being of a young adult person.

The purpose of the LBA study was to study the lifestyle of young adults and to evaluate the effect of different lifestyle habits on biomarkers known to influence the risk of CVD. We, and others, have previously published data showing that young adult women and men with high CRF have more beneficial biomarkers for lipid- and glucose metabolism compared to those with low CRF [13, 14]. The positive effects of high CRF on the heart and the circulation are well known, stroke volume increases and the resting heart rate (HR) becomes lower [15]. In line with this data it has been shown that those who reach the physical activity guidelines recommendations of 30 min per day of moderate- and vigorous physical activity (MVPA) have a reduced risk of suffering from CVD in the future [16, 17]. Overweight and obesity is increasing worldwide and in all age groups [18]. Obesity was present in 20% of the study participants in the LBA-study [19]. In addition, when markers such as blood pressure, high-density lipoprotein cholesterol (HDL-C) and homeostasis model assessment of insulin resistance (HOMA-IR) where used to create the Wildman CVD risk score, 12% of the young adult women and men were classified as being at risk for future CVD [13]. This is in line with the result from a study in the USA that shows high prevalence of CVD risk in college students, aged 18–24 years, and the need for screening young adults at risk for CVD was pointed out by the authors [20]. The high number of young adults at risk for future CVD indicates the need for increased knowledge of the lifestyle of young adults, and the need to find easy non-invasive markers to detect those at risk - young adults who needs counselling about a healthy lifestyle.

### Aims

The aims of the present study were to evaluate sleep habits of the young adult women and men, participating in the LBA study, and to compare the importance of sleep habits and other lifestyle habits with clinically relevant biomarkers for CVD. An additional aim was to find easy and reliable non-invasive biomarkers to detect young adults with increased risk of developing CVD later in life.

## Methods

In the cross-sectional Lifestyle, Biomarkers and Atherosclerosis (LBA) study conducted at Örebro University, Sweden, young, self-reported healthy adults were examined for early signs of atherosclerosis. Recruitment was done by advertisement at the university, in local newspapers and in social media. For more detailed information, see [13]. The Uppsala ethics committee approved the study design (DNR: 2014/224). All participants gave their written consent to participate, and were informed that they could terminate their participation at any time.

### Body fat in percent and BMI

Height, weight and percentage of body fat, were measured with the study participants in a fasting state. Height was measured to the nearest 0.5 cm with a wall mounted stadiometer. The participants stood straight without shoes, feet together and with the arms extended along the body. Weight was measured to the nearest 0.1 kg, and percentage of body fat was calculated, using an impedance body composition analyser (Tanita Co, Tokyo, Japan). The study participants stood barefoot on the conductive equipment, holding metal handles, according to the manufacturer’s guidelines. Adjustments were made, as recommended by the instruction manual [21], for 1 kg off clothes and a standard setting was used. The participant’s age, sex and height were registered in the body composition analyser. Data on body weight and body fat (%) was collected from the analyser and BMI (kg/m^2^) was calculated by the equipment.

### Serum biomarkers

Blood samples were collected from the participants after 20 min of rest, following an 8-12 h fasting period. The area for venepuncture was cleaned and the venepuncture was performed with a 21 gauge butterfly needle (Greiner Bio-One International GmbH, Vacuette®, Rainbach im Mühlkreis, Austria). After the blood collection, the tubes (BD Vacutainer; BD AB, Stockholm, Sweden) were gently inverted several times. Blood for analysing high-density lipoprotein cholesterol (HDL-C) and low-density lipoprotein cholesterol (LDL-C) was collected into lithium-heparin tubes, and plasma was obtained by centrifugation for 8 min at 2000×g in room temperature. A citric acid-citrate-NaF tube was used to collect blood to analyse glucose. Serum for insulin analysis was obtained by collecting blood in a standard serum tube with clot activating substances. The blood samples were allowed to clot for at least 30 min before centrifugation for 8 min at 2000×g in room temperature. All tubes were afterwards placed in +4^o^ C until transportation and analyses at the accredited clinical chemistry laboratory at Örebro university hospital.

HDL-C, LDL-C and glucose (mmol/L) were analysed on an Ortho Clinical Diagnostics TM (Clinical Chemistry instruments, Vitros 5,1TM FS, Raritan, New Jersey, U.S.A.). The method was dry chemistry (colorimetric method) according to the manufacturer’s (Orthos) instructions. Insulin (mU/L) was analysed on an Architect instrument (i2000SR from Abbott, Illinois, U.S.A.), with their reagent according to their instructions on antibody-based technologies.

Homeostasis model assessment of insulin resistance (HOMA-IR) was calculated by using the mathematical equation by Matthews, (insulin (mU/ml)*glucose (mmol/L)/22.5). In the present study the HOMA-IR value was used as a measure of insulin resistance [22].

### Mean arterial pressure (MAP) and resting heart rate (HR)

Blood pressure and heart rate were measured after approximately 10–15 min rest using a digital automated device (GE Healthcare, Dinamap V100, Buckinghamshire, UK) with Dura-Cuf (GE Medical Systems, GE Criticon Dura-cuf, Milkaukee, WI, US). The brachial blood pressure was measured in the left arm with the participants in a supine position. At least three measurements were done with two minutes intervals. When the difference between the latest systolic pressures was less than 5 mmHg the measurement was ended. The results for MAP and resting heart rate were reported as an average of the two latest results.

### Risk for CVD - Wildman score

The Wildman risk score [23, 24] has been used to classify participants at risk for CVD. Individuals with two or more of the following characteristics were classified as being at risk according to Wildman; elevated blood pressure (130/85 mmHg), elevated triglycerides (≥1.70 mmol/L), decreased HDL-C (women < 1.30 mmol/L, men < 1.04 mmol/L), elevated glucose (≥5.6 mmol/L), Insulin resistance (HOMA-IR > 2.52), and elevated hs-CRP (> 5.07 mg/L).

### Moderate- and vigorous physical activity (MVPA)

The subject’s physical activity (PA) was measured with an accelerometer (ActiGraph, model GT3X+, Pensacola, FL, USA). The participants were instructed to wear the accelerometer on an elastic belt around their waist in the middle of their lower back during all waking time for one week. The accelerometer data was processed and analysed with the Actilife software (ActiLife, version 6.13.3, ActiGraph, Pensacola, FL, USA). The accelerometer was initialized with a sampling frequency of 30 Hz, and the vertical axis acceleration with 60-s epoch was used. Non-wear time was defined by an interval of at least 60 min of 0 counts per minute with an allowance for maximum 2 min of counts between 0 and 100. The participants included in the analyses needed at least 10 h or more of wear time on the days of measurement [25]. The cut-off point used to define moderate- and vigorous physical activity (MVPA) was ≥2020 counts [26]. Time spent in MVPA, is presented as minutes/day. MVPA categories were defined as follow: ≥ 30 min/day and < 30 min/day.

### Cardiorespiratory fitness (CRF) measured as maximal oxygen uptake (VO_2_ max)

To measure CRF, a modified two point submaximal Åstrand exercise test was performed and maximal oxygen uptake (VO_2_ max) was calculated [13, 27]. The exercise test was done on a Monark 939E (Monark Sports & Medical, Monark 939E, Vansbro, Sweden) with ECG registration to monitor heart rate (Cardiolex, EC Sense, Solna, Sweden). The exercise test started on an individually adjusted level between 50 and 100 W depending on the participants’ exercise habits. The cycling continued until the first steady-state level was reached, and then the workload was increased to reach next steady-state level. The exercise test ended when the participant reached steady state at two workload levels, with a heart rate above 130 beats/min on the first level and above 150 beats/min on the second level. The estimated VO_2_ max was calculated by the heart rates at the two steady-state levels and the expected oxygen consumption per work rate, by using the equation of the straight line. Maximal heart rate was estimated through the formula 220 - age in years. The study participants were categorized as having low, normal or high VO_2_ max according to European reference values [28]. Categories for VO_2_ max for women were: low (≤ 30 ml/kg/min), normal (30.1–39.9 ml/kg/min), and high (≥ 40 ml/kg/min) VO_2_ max. Categories for men were: low (≤ 40 ml/kg/min), normal (40.1–49.9 ml/kg/min), and high (≥ 50 ml/kg/min) VO_2_ max.

### Handgrip strength

Muscle strength was measured with a Dynamometer (Fabrication Enterprices inc, Baseline® HiRes™ hydraulic hand dynamometer, Irvington. NY, US). First, the hand size was measured with a measuring tape, and the dynamometer was adjusted to fit the size of the hand [29]. Handgrip strength was measured in the dominant hand. The participants sat with the arm at a 90° angle. All participants first performed one practice test, before three measurements were taken, with one minute rest between each messurment. The result was calculated as an average of the three measurements. The study participants were categorized as having low, normal or high muscular strength according to sex specific reference values [30]. Limits for handgrip strength categories for females were: low ≤22 kg, normal 22.1–34.9 kg, and high ≥35 kg. For males the corresponding levels were: low ≤37 kg, normal 37.1–56.9 kg, and high ≥57 kg.

### Sleep habits

All study participants filled in a validated computerized questionnaire about their general physical and mental health [31]. In the questionnaire two questions on sleep duration and experienced quality of sleep were added. The first question concerned sleep duration and the participants were asked if they sleep on average, less than 6 h, 6 to 7 h or more than 7 h per night. In the second question the participants were asked, A: Do you experience good quality of sleep? B: Do you experience troubled sleep with several awakenings per night? C: Do you often have difficult falling asleep? In the second question the participants could choose to answer yes or no.

### Food habits

The participants also filled in the computerized food frequency questionnaire “Food habit choice” from the Swedish national food agency [32]. The questions were based on the dietary recommendations from the Swedish national food agency [33], and the results were based on the total response to the questionnaire and presented as a score from 1 to 12 points. Participants with 1 to 4 points were considered to have unhealthy food habits. Participants having 5 to 8 points were considered to have healthy food habits with potential for improvement, and participants having 9–12 points were considered as having food habits according to the recommendations from the Swedish national food agency.

### Statistics

Statistical calculations were performed using Excel (2016 for windows) (Microsoft Co, Redmond, WS, USA) and IBM SPSS Statistics, version 25 for Windows (IBM Corp, Armonk, NY, USA). The Kolmogorov Smirnov and the Shapiro Wilk test were used to check all variables for normal distribution. A two-sided independent Student’s *t*-test was used to compare means of basic characteristics and lifestyle factors between women and men. Equal variances were assumed. Comparison of means between sex in the non-parametric qualitative variables (MVPA and food habits score) were analysed by Mann-Whitney U test. Pearson’s correlation coefficient (*r*) was used to study associations between variables.

To study the relationship between variables, linear regression was used. The odds ratio (OR) of belonging to the category “At risk according to Wildman” was analysed by logistic regression, first by entering the lifestyle factors one by one and thereafter by entering all lifestyle factors in the same model. In the logistic regression analyses the variables were coded in an “increased risk” order i.e. for VO_2_ max, sleep duration, food habits, and handgrip strength (at the levels high/normal/low), and for MVPA (at the levels high/low). The probability of being at risk according to Wildman risk score, was expressed as exponential beta, Exp (B). The levels of significance have been expressed as *P* < 0.05 = *, *P* < 0.01 = ** or *P* < 0.001 = ***. To illustrate correlations and quartiles Excel for windows was used.

## Results

In total 829 participants (574 women and 255) men filled in the validated computerized questionnaire about their general physical and mental health, with questions about sleep habits, and were included in the analyses. From the 834 study participants in the LBA study, five questionnaire registrations were missing due to technical reasons. There are also some missing values for some of the biomarkers and lifestyle factors. HDL-C is missing for five participants and LDL-C for six participants. HOMA-IR is missing for 25 participants and CRF for 13 participants. Basic characteristics of biomarkers and lifestyle factors of the study participants are presented divided by sex, with expected significant differences between women and men presented (Table 1).

**Table 1:** Basic characteristics of biomarkers and lifestyle factors, divided by sex, for the 834 study participants in the LBA study. *P*-value and significance indicate differences between women and men

	Women (*N* = 574)	(SD)	Men (*N* = 255)	(SD)	*P*-value	Significance
	(mean)		(mean)			
Age (years)	21.8	1.9	22.0	2.0	0.221	NS
Biomarkers						
BMI (kg/m^2^)	22.4	3.6	23.4	3.1	0.606	NS
Body fat (%)	28.0	6.6	14.8	5.6	0.016	*
HDL cholesterol (mmol/L)	1.4	0.4	1.2	0.3	P < 0.001	***
LDL cholesterol (mmol/L)	2.3	0.7	2.3	0.7	0.665	NS
Resting HR (beats/min)	66	10.4	63	10.1	0.403	NS
MAP (mmHg)	81.0	7.4	87.1	8.9	0.001	**
HOMA-IR	1.8	1.1	1.8	0.9	0.505	NS
Lifestyle factors						
CRF – VO_2_ max (ml/kg/min)	37.8	8.5	42.9	9.9	0.002	**
Handgrip strength (kg)	34.4	6.5	53.1	10.1	P < 0.001	***
	(median)	(Q1-Q3)	(median)	(Q1-Q3)		
MVPA (min/day)	44	30–58	44	29–59	0.978	NS
Food habits (points)	6.0	5–7	6.0	4–7	0.062	NS

*BMI* body mass index, *Body fat (%)* percentage of body fat, *HDL-C* high-density lipoprotein cholesterol, *LDL-C* low-density lipoprotein cholesterol, *Resting HR* resting heart rate, *MAP* mean arterial pressure, *HOMA-IR* homeostasis model assessment of insulin resistance, *MVPA* moderate- and vigorous intensity physical activity and *CRF* cardiorespiratory fitness measured as estimated maximal oxygen uptake, VO_2_ max

Data are presented as mean and SD. Differences between women and men were analysed by unpaired Student’s t-test. Food habits and MVPA are presented as median and interquartile range (Q1-Q3). Differences in food habits and MVPA between women and men were analysed by Mann-Whitney U test

Levels of significance were set to *P* < 0.05 = *, *P* < 0.01 = ** or *P* < 0.001 = *** and NS, not significant

The results of the questions on sleep duration and experienced sleep quality are presented in percent for the total population, and divided by sex. In total 604 participants answered that they experienced good sleep quality. Of the remaining, 114 experienced troubled sleep with several awakenings per night and 110 had difficulties falling asleep. In total 73% of the participants reported that they experienced good sleep quality, and the remaining 27% that they had troubled sleep with several awakenings per night or often had difficulty falling asleep. 438 participants answered that they usually slept more than seven hours, 366 slept six to seven hours and 25 slept less than six hours per night (Table 2).

**Table 2:** Summary of the results on questions about sleep

Question	Answer alternatives	Total	Women	Men
1	More than seven hours	53%	54%	50%
1	Six to seven hours	44%	43%	46%
1	Less than six hours	3%	3%	3%
2	Good	73%	72%	74%
2	Anxious with several awakenings per night	14%	15%	10%
2	Often difficult to fall asleep	13%	12%	16%

1. How many hours per night do you usually sleep? 2. How do you experience the quality of your sleep?

BMI, body fat (%), HOMA-IR and risk factors for CVD (Wildman score) were significantly correlated to sleep duration, indicating that, in this population, less sleep per night was associated to higher BMI, more body fat (%), less advantageous HOMA-IR values and an increased risk of future CVD (Table 3). None of the biomarkers presented in Table 3, were significantly correlated with experienced quality of sleep, but “sleep in hours” and “quality of sleep” where significantly correlated to each other, (*r* = 0.26, *P* < 0.01).

**Table 3:** Bivariate correlation analyses of the result on the questions about sleep on chosen biomarkers

	Pearson’s correlation coefficient (*r*)	Sleep in hours per night Significance	Pearson’s correlation coefficient (*r*)	Quality of sleep Significance
BMI (kg/m^2^)	0.148	**	0.047	NS
Body fat (%)	0.077	*	0.023	NS
HDL cholesterol (mmol/L)	−0.053	NS	−0.004	NS
LDL cholesterol (mmol/L)	−0.007	NS	−0.001	NS
Resting HR (beats/min)	0.018	NS	0.035	NS
MAP (mmHg)	0.063	NS	−0.008	NS
HOMA-IR	0.079	*	0.022	NS
Risk for CVD - Wildman	0.092	**	0.031	NS

*BMI* body mass index, *Body fat (%)*percentage of body fat, *HDL-C* high-density lipoprotein cholesterol, *LDL-C* low-density lipoprotein cholesterol, *Resting HR* resting heart rate, beats/min, *MAP* mean arterial pressure, *HOMA-IR* homeostasis model assessment of insulin resistance and Risk for *CVD* cardiovascular disease

Levels of significance were set to *P* < 0.05 = *, *P* < 0.01 = ** or *P* < 0.001 = *** and NS, not significant

The lifestyle factors CRF, measured as VO_2_ max, and MVPA were correlated to sleep habits. VO_2_ max was significantly negatively correlated to both “sleep in hours” and “quality of sleep”, (*r* = − 0.07 and *r* = − 0.09, *P* < 0.05). MVPA was correlated to the experienced quality of sleep (*r* = − 0.10, *P* < 0.01). Handgrip strength and food habits were not correlated to sleep duration or sleep quality.

The OR of belonging to the category “At risk according to Wildman” were statistically significant for CRF, sleep in hours per night, MVPA, and food habits, but not for handgrip strength, Table 4. In Table 5 the independent variables were analysed in a multiple model and showed that CRF and sleep in hours per night remained significant, with MVPA as the third most important modifiable lifestyle factor, closely followed by food habits and handgrip strength.

**Table 4:** Logistic regression with CVD risk (*at risk or not at risk, measured by Wildman score*) as dependent variable, and modifiable lifestyle factors as independent variables entered one by one

Lifestyle factors	Exp (B)	*P*-value	Significance
CRF - VO_2_ max (ml/kg/min)	2.053	< 0.001	***
Sleep in hours per night	1.760	0.002	**
MVPA (min/day)	1.692	0.025	*
Food habits (points)	1.552	0.024	*
Handgrip strength (kg)	1.326	0.143	NS

*CRF* cardiorespiratory fitness measured as estimated maximal oxygen uptake, *VO*_*2*_ *max and MVPA* moderate- and vigorous intensity physical activity

Levels of significance were set to *P* < 0.05 = *, *P* < 0.01 = ** or *P* < 0.001 = *** and NS, not significant

**Table 5:** Multiple logistic regression with CVD risk (*at risk or not at risk, measured by Wildman score*) as dependent variable, and modifiable lifestyle factors as independent variables entered in the same model, to create the odds ratio (OR)

Lifestyle factors	Exp (B)	*P*-value	Significance
CRF - VO_2_ max (ml/kg/min)	1.792	< 0.001	***
Sleep in hours per night	1.617	0.014	*
MVPA (min/day)	1.472	0.112	NS
Food habits (points)	1.351	0.133	NS
Handgrip strength (kg)	1.074	0.719	NS

*CRF* cardiorespiratory fitness measured as estimated maximal oxygen uptake, *VO*_*2*_ *max and MVPA* moderate- and vigorous intensity physical activity

Levels of significance were set to *P* < 0.05 = *, *P* < 0.01 = ** or *P* < 0.001 = *** and NS, not significant

**Fig. 1:**
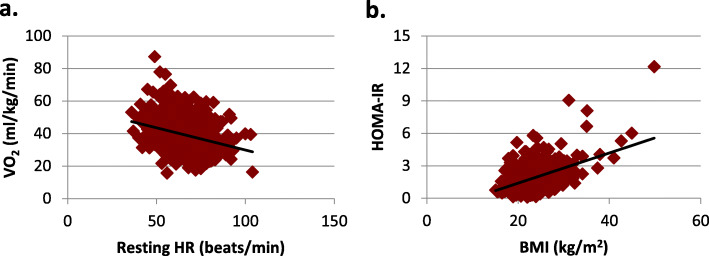
**a**. Illustration of the correlation between cardiorespiratory fitness measured as estimated maximal oxygen uptake, VO_2_ max and resting heart rate (HR), (r = − 0.31, *P* < 0.01). **b**. Illustration of the correlation between insulin resistance measured as homeostasis model assessment of insulin resistance (HOMA-IR) and body mass index (BMI), (r = 0.48, *P* < 0.01).

**Fig. 2:**
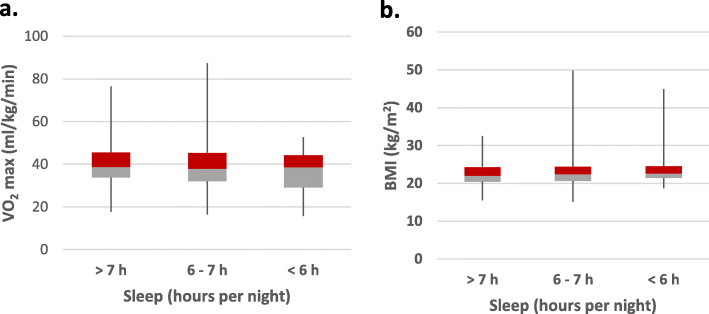
**a.** The figure shows the relation of sleep duration (hours per night) in quartiles and cardiorespiratory fitness measured as estimated maximal oxygen uptake, VO_2_ max. **b.** The figure shows the relation of sleep duration (hours per night) and body mass index (BMI). In both figures the box shows the 25–75 percentiles and the whiskers the entire span from 0 to 100 percentiles. The colours change expresses the median line

In the search of non-invasive, easy and reliable biomarkers to detect young adults with increased risk of developing CVD later in life, a negative correlation between CRF measured as VO_2_ max (ml/kg/min) and resting HR (*r* = − 0.31, *P* < 0.01) was illustrated (Fig. 1a). When split by sex, this negative correlation was *r* = − 0.30, *P* < 0.01 for women, and *r* = − 0.26, *P* < 0.01 for men. VO_2_ max (ml/kg/min) includes body weight and there were significantly negative associations between VO_2_ max and BMI (*r* = − 0.24, *P* < 0.01), and between BMI and resting HR (*r* = 0.07, *P* < 0.05). A correlation between HOMA-IR, a risk marker for diabetes, and BMI (*r* = 0.48, *P* < 0.01) was illustrated (Fig. 1b). When split by sex, this correlation was (*r* = − 0.53, *P* < 0.01) for women, and (*r* = − 0.33, *P* < 0.01) for men. To demonstrate the effect of sleep duration (hours per night) on the biomarkers BMI and resting HR, quartiles of BMI and resting HR were created for the different sleep durations, less than seven hours, 6 to 7 h and more than 7 h (Fig. 2 a and b).

## Discussion

In total 27% of the study participants answered that they had difficulties falling asleep or experienced troubled sleep with several awakenings per night. The observed correlation between sleep quality and sleep duration indicates that it is important to take the problem seriously. Especially considering that shorter sleep duration was associated to higher BMI, body fat (%), HOMA-IR and CVD risk (Table 3).

The importance of sleep in adolescents and young adults has been pointed out in the review by Bruce et al. [9]. The relationship between sleep problems and increased body weight, have also consistently been pointed out, especially in children and young adults [8, 34]. Overweight in its turn increases the risk of type 2 diabetes and CVD, making sleep habits an important lifestyle factor to consider in the preventive work against CVD. The probable mechanisms behind the increased BMI, among subjects with shorter sleep duration, which was present in this study, are changes in the appetite regulating hormones leptin and ghrelin, and the increased opportunity to eat. Another plausible explanation is reduced energy expenditure due to sleep deprivation, and that sleep deprivation perhaps may lead to a more sedentary lifestyle [35].

The possible risk of sedentarism due to sleep problems is supported by the results from the present study, showing that the participants with sleep quality problems spend less time per day in MVPA compared to the study participants with good sleep. Furthermore, participants with high CRF had better sleep quality and longer sleep duration compared to the less well-trained study participants. In this population of young adults strength training was popular, but handgrip strength was not associated with sleep habits or CVD risk. If regular cardiorespiratory training leads to a better sleep quality and longer sleep duration or if sleep deprivation leads to a more sedentary lifestyle is an open question, but regardless of which comes first, physical activity and sleep habits are important in early CVD prevention.

In the present study sleep duration was expressed as less than 6 h per night, 6 to 7 h per night, or more than 7 h per night. The majority of the study participants slept 6 to 7 h per night, or more than 7 h per night, with only a few percent sleeping less than 6 h per night. In a dose-response meta-analysis of prospective studies a U-shaped relationship between sleep duration and type 2 diabetes risk was shown [36]. The relation showed that both short and long sleep duration is associated with increased risk, and that 7 to 8 h of sleep per night is associated with the lowest risk of type 2 diabetes. It was concluded that appropriate sleep duration is important to prevent or delay type 2 diabetes. The association between sleep in hours per night and insulin resistance measured as HOMA-IR and risk for CVD (Wildman score), in the present study, shows that the association is also present in young adults.

In the present study, 53% of the participants answered that they slept more than 7 h per night. This implies that almost half of the study participants did not have sufficient sleep duration to reduce the type 2 diabetes risk, in the most optimal way. In general, sleep habits have changed in adolescents and young adults due to the 24-h culture of connectivity and media consumption [37]. This may result in young adults staying awake later, with reduced sleep quality and duration. Many physicians encounters young adults with complains or conditions that are related to poor sleep [9]. Poor sleep in adolescents and young adults can result in long term sleep problems with an impact on adult life [38]. The authors of the review, “Sleep in adolescents and young adult” [9] concluded that it is important to identify and manage sleep problems before long-term consequences develop.

The result from the multiple logistic regression analysis, aiming to rank the influence of lifestyle factors on CVD risk and create the odds ratio, showed that sleep duration was the second most important lifestyle factor (Table 5). Even if the purpose of the study was to evaluate the effect of sleep habits on CVD risk, by far the most important modifiable lifestyle factor, with regard to CVD risk was CRF. The non-multiple logistic regression analyses, with the independent variables entered one by one, showed that in addition to CRF and sleep duration, MVPA and food habits are also important with respect to CVD risk (Table 4). The WHO recommendations on PA addresses the entire age range between 18 and 64 years [39]. It can be argued that more MVPA is needed in this age group (18–25 years) and that the recommendations should be different for different age groups. The Wildman CVD risk score used in this study is created by biomarkers such as blood pressure, HDL-C and HOMA-IR showing the importance of biomarkers for lipid- and glucose metabolism in CVD prevention also in this age group.

A decline of 11% in CRF in the Swedish working population has been shown, and during the period from between 1995 and 2017, the proportion of people with low CRF increased from 27 to 46% [40]. The decline was most pronounced in men and younger people, while men with a short education were pointed out as especially vulnerable [40]. In the present study a majority of the participants were women, and students at the university were overrepresented. This indicates that the results from the present study may even underestimate the severity of the problem with unhealthy sleep habits and low CRF in young Swedish adults. Sleep problems with short sleep duration in combination with reduced cardiovascular fitness, in young adults, is a serious health issue especially considering that a large cohort study showed that CVD mortality was inversely related to CRF measured as VO_2_ max in both women and men [2].

In search of non-invasive, easy and reliable biomarkers to detect young adults with increased risk of developing CVD later in life, BMI and resting HR in combination with questions about sleep patterns are suggested. BMI is already used in practice and the correlation to HOMA-IR in the present study (Fig. 1b) in combination with previously shown relationships between food habits and HOMA-IR [41], indicates that BMI is a reliable biomarker of type 2 diabetes and CVD risk in this age group.

The relationship between CRF measured as VO_2_ max and resting HR, in this population, is illustrated in Fig. 1a. The relation has previously been shown, in articles, for example from the Mayo Clinic [42, 43]. The authors concluded that lower cardiorespiratory fitness levels and higher resting HR are linked to higher CVD and all-cause mortality. In line with these findings, Jensen et al. mention the causal link between resting HR and longevity, and that it should be expected that health care providers give advice regarding HR to the public [44]. It is also suggested to simplify cardiovascular risk estimation by using resting HR. The authors claim that inclusion of resting HR in simple systems can potentially enhance cost-effectiveness and accessibility in risk estimation [45]. However, the articles present results from studies with older subjects with health issues, and not healthy young adults. Another study that has examined the relationship between resting HR and CVD risk is the HELENA study. They state that resting HR is not a good predictor of a clustered cardiovascular risk score in adolescents [46]. In the HELENA study the inclusion criteria was 12.5–17.5 years. This is an age when many adolescents go through puberty with changes occurring in the body that may affect the relationship, this is not the case in young adults, aged 18–25 years.

In Sweden many young adults, especially young women, experience stress [47]. Stress problems were not the scope of the present article, but they are also a risk factor for CVD. Since resting HR reflects the sympathetic nerve activity, we think that stress problems will be captured in the measurements of resting HR, or as sleep problems. It is important to remember, in the screening of young adults at risk for CVD, to add adequate questions about sleep habits. One such example of a tool to identify sleep problems is the BEARS screening tool with questions about sleep habits categorised as; bedtime problems, daytime sleepiness, awakenings during the night, regularity and duration of sleep and sleep disordered breathing [9].

The strength of the present study is that the large amount of data on biomarkers and lifestyle factors, gives a wider perspective on the influence of sleep habits in young adults. The study participants, however, may not be entirely representative of young Swedish adults in general. It was difficult to recruit men, and despite persistent efforts to include as many men as women, only 30% of the participants were men. Another previously mentioned problem was that many of the participants were studying at the university and had an interest in health. This could potentially have led to participants already having healthier lifestyle habits, as compared to young adults in general. It may also be argued that the large number of participants in the present study makes it easy to get low *P*-values. Nevertheless, we found many young adults who clearly need help in changing their lifestyle. The present study shows that sleep problems, low CRF and increased CVD risk is common among young adult university students, and the results of the study underline the importance of addressing this health issue.

## Conclusion

In the present, study sleep quality and sleep duration in young adults, aged 18–25 years, were examined. In total 27% of the participants experienced sleep problems. Sleep quality and duration were correlated, and shorter sleep duration was associated with higher BMI, body fat (%), HOMA-IR and CVD risk. The modifiable lifestyle factor with the highest odds ratio for CVD risk, measured with the Wildman score, was CRF. Sleep duration was the second most influential modifiable lifestyle factor for this age group and more important than MVPA and food habits.

Decreased sleep duration in combination with decreased CRF, in the young adult population, is a serious health issue that needs to be taken seriously. In search of non-invasive, easy and reliable biomarkers to detect young adults with increased risk of developing CVD later in life, BMI and resting HR in combination with questions about sleep patterns are suggested.

## Data Availability

All raw data are available at Örebro University, contact person Anita Hurtig-Wennlöf.

## Abbreviations

AFA: Asset Management Arm
BMI: Body mass index
CVD: Cardiovascular diseases
CRF: Cardiorespiratory fitness
Exp (B): Exponential beta
HDL-C: High-density lipoprotein cholesterol
HOMA-IR: Homeostasis model assessment of insulin resistance
LBA study: Lifestyle, Biomarkers, and Atherosclerosis study
LDL-C: Low density lipoprotein cholesterol
MAP: Mean arterial pressure
MVPA: Moderate- and vigorous physical activity
NS: Not significant
OR: Odds ratio
PA: Physical activity
SD: Standard deviation
VO_2_ max: Maximal volume of oxygen uptake
